# Prospective study of once-daily accelerated partial breast irradiation using 3-dimensional conformal external beam radiotherapy for Japanese women: 12-year outcomes, toxicity, and cosmesis

**DOI:** 10.1007/s12282-024-01650-x

**Published:** 2024-12-04

**Authors:** Kana Takahashi, Yoshikazu Kagami, Ryoichi Yoshimura, Madoka Morota, Naoya Murakami, Satoshi Nakamura, Hiroyuki Okamoto, Ayaka Nagao, Madoka Sakuramachi, Tairo Kashihara, Tomoya Kaneda, Koji Inaba, Kae Okuma, Yuko Nakayama, Jun Itami, Hiroshi Igaki

**Affiliations:** 1 Department of Radiation Oncology, National Cancer Center Hospital, 5-1-1 Tsukiji, Chuo-ku, Tokyo, 104-0045 Japan; 2 Department of Radiation Oncology, Showa University, 1-5-8 Hatanodai, Shinagawa-ku, Tokyo, 142-8666 Japan; 3 Department of Radiation Therapeutics and Oncology, Tokyo Medical and Dental University, 1-5-45 Yushima, Bunkyo-ku, Tokyo, 113-8510 Japan; 4 Department of Radiation Oncology, Showa University Koto Toyosu Hospital, 5-1-38 Toyosu, Koto-ku, Tokyo, 135-0061 Japan; 5 Department of Radiation Oncology, Juntendo University Graduate School of Medicine, 3-1-3 Hongo, Bunkyo-ku, Tokyo, 113-8431 Japan; 6 Division of Radiation Safety and Quality Assurance, National Cancer Center Hospital, 5-1-1 Tsukiji, Chuo-ku, Tokyo, 104-0045 Japan; 7 Shin-Matsudo Accuracy Radiation Therapy Center, Shin-Matsudo Central General Hospital, Shin-Matsudo 1-380, Matsudo-City, Chiba 270-0034 Japan

**Keywords:** APBI, Once-daily, 3D-CRT, Breast cancer, Japanese women

## Abstract

**Background:**

To analyze in a prospective study the long-term safety and efficacy of 3-dimensional conformal radiotherapy (3D-CRT) to deliver accelerated partial breast irradiation (APBI) for Japanese women with early breast cancer.

**Methods:**

Breast cancer patients with pathological tumor size ≤ 3 cm, age ≥ 20 years, lumpectomy with at least a 5 mm margin, and ≤ 3 positive axillary nodes were eligible. APBI was delivered by 3D-CRT at a dose of 38.5 Gy in 10 fractions over 10 days. The primary endpoints were the frequency and severity of acute and late radiation toxicities, and secondary endpoints were local control, survival, and cosmesis. The sample size was determined based on the incidence of ≥ grade 3 acute and late radiation toxicities, which required 71 enrollments.

**Results:**

Between 2008 and 2010, 73 patients enrolled in this trial. Twelve patients (16%) had 1–3 lymph node metastases. At a median follow-up of 12.6 years (range: 2.7–13.9 years), there were no cases of grade ≥ 3 acute or late toxicity. There were 4 ipsilateral breast tumor recurrence (IBTR) events: 12-year IBTR incidence was 4.4%. The difference in the incidence of IBTR between node-negative and node-positive patients was marginal (1.9% vs. 16.7%, p = 0.055). The majority of patients (94.4% at 2 years, 89.3% at 10 years after enrollment) had excellent/good cosmesis.

**Conclusions:**

APBI delivered with 3D-CRT is a feasible treatment option for Asian females, but it was indicated that node-positive status might increase IBTR risk.

**Supplementary Information:**

The online version contains supplementary material available at 10.1007/s12282-024-01650-x.

## Introduction

Accelerated partial breast irradiation (APBI) has increasingly been offered as an alternative to conventional external beam whole breast irradiation (WBI) following lumpectomy in selected early-stage breast cancer patients [1]. Different treatment techniques and dose fractionations have been utilized in APBI [2]. The majority of trials have indicated similar treatment outcomes with APBI in carefully selected patients. However, determining the most effective schedule that achieves the optimal balance between patient preference, local control, toxicity, and cosmesis remains an issue.

APBI is rarely used in Japan. One reason for this is the difficulty in target identification caused by oncoplastic surgery, which is generally adopted in Japan. Secondary: APBI is often unsuitable for Asian females because they generally have smaller breast sizes than Western females.

In 2008, we conducted a study (umin0000003405) to examine the use of once-daily APBI with 3-dimensional conformal radiation therapy (3D-CRT). We carried out this trial to test the safety and efficacy of APBI delivered with 3D-CRT, even for Japanese females. We present 12-year outcomes in terms of local control, treatment-related toxicities, and cosmesis.

## Materials and methods

### Patient selection

Eligibility criteria were as follows: pathological tumor size ≤ 3 cm; invasive ductal carcinoma and special types including medullary, papillary, mucinous, invasive lobular, or apocrine histologies (other than squamous cell carcinoma) of the breast as well as ductal carcinoma in situ; unifocal lesion; age ≥ 20 years; lumpectomy with negative surgical margins (defined as at least a 5 mm margin); lumpectomy cavity identifiable on planning computer tomography (CT) with titanium clips; and ≤ 3 positive axillary nodes (proved by sentinel lymph node biopsy and/or axillary dissection). Exclusion criteria included the following: prior history of radiotherapy for ipsilateral breast; neoadjuvant chemotherapy or hormonal therapy; bilateral breast cancer; concomitant malignancies; systemic lupus erythematosis, scleroderma, dermatomyositis; pregnancy or lactation.

This prospective study was approved by the Institutional Review Board of the National Cancer Center on October 22, 2007 (reference number: 19–042), and all enrolled patients gave their written informed consents before being registered in the study.

After the start of this study, the American Society of Radiation Oncology (ASTRO) Consensus Statement of APBI was published in 2009, and an update of the ASTRO Consensus Statement was published in 2017 [3, 4] (Online Resource1). We considered the ASTRO suitability group to be a prognostic factor and classified the cases into three groups in the Results section.

### Simulation and treatment planning

Treatment planning and delivery were performed with the patient in the supine position. Patients were first imaged (3-mm CT slice thickness) with both arms raised over the head. The normal breast volume was defined by applying radiopaque wires to define the volume that would have been treated by classic whole-breast tangents. The lumpectomy cavity, ipsilateral and contralateral breasts, bilateral lungs and heart were delineated on each CT slice. The clinical target volume (CTV) was defined as an expansion of the lumpectomy cavity including surgical clips with 1 cm in all directions minus chest wall and 2 mm thick tissue under the skin. An isotropic 1 cm margin was added to the CTV to obtain the planning target volume (PTV). For evaluating the dose of the PTV, PTV_EVAL was generated from the PTV, excluding the lung, chest wall and 2 mm thick tissue under the skin.

For each patient, treatment was delivered by 3D-CRT using 2–4 coplanar or noncoplanar fields with 4 or 6 MV photons. Intensity modulated radiation therapy was not allowed. A total of 38.5 Gy in 10 fractions were prescribed to the ICRU point (located centrally in the PTV on the beam axes at their intersection). Single fraction per day, each 3.85 Gy, were given in 10 consecutive working days. The dose-volume constraints were based on National Surgical Adjuvant Breast and Bowel Project (NSABP) B39/Radiation Therapy Oncology Group (RTOG) 0413 protocols, but we changed the constraint of PTV_EVAL as in Table 1, because it was easy to satisfy the original dose limitation of PTV_EVAL and higher part of the PTV_EVAL dose could be appropriately evaluated with D90_PTV_EVAL_ (Table 1).

**Table 1:** Dose-volume constraints

	Allowed value	Evaluation
**PTV parameter**		
D90_PTV_EVAL_	≥ 90% of the prescribed dose	DVH of PTV_EVAL
**Original PTV parameter of NSABP B39/ RTOG 0413**		
V90_PTV_EVAL_	≥ 90% of PTV_EVAL	DVH of PTV_EVAL
**OAR parameters**		
Ipsilateral breast maximum	≤ 120% of prescribed dose	Point dose
V50_ipsi_breast_	< 60% of breast volume	DVH of ipsilateral breast
V100_ipsi_ breast_	< 35% of breast volume	DVH of ipsilateral breast
Contralateral breast maximum	< 3% of prescribed dose	Point dose
V30_ipsi_lung_	< 15% of lung volume	DVH of ipsilateral lung
V5_contra_lung_	< 5% of lung volume	DVH of contralateral lung
V5_heart_ (right-sided lesion)	< 5% of heart volume	DVH of heart
V5_heart_ (left-sided lesion)	< 40% of heart volume	DVH of heart

*PTV* planning target volume, *PTV_EVAL* planning target volume for evaluation, *NSABP* the National Surgical Adjuvant Breast and Bowel Project, *RTOG* Radiation Therapy Oncology Group, *OAR* organ at risk, *ipsi_breast* ipsilateral breast, *ipsi_lung* ipsilateral lung, *contra_lung* contralateral lung, *D90*_*V90PTV_EVAL*_ the minimun coverage dose of 90% of the PTV_EVAL, *V90*_*PTV_EVAL*_ the volume of the PTV_EVAL covered by 90% of the prescribed dose, *V50, V100*_*ipsi_breast*_ the volume of the ipsilateral breast covered by 50% or 100% of the prescribed dose, *V30*_*ipsi_lung*_ the volume of the ipsilateral lung covered by 30% of the prescribed dose, *V5*_*contra_lung*_ the volume of the contralateral lung covered by 5% of the prescribed dose, *V5*_*heart*_ the volume of the heart covered by 5% of the prescribed dose

### Dose-fractionation design

We adopted a dose-fractionation of 38.5 Gy in 10 fractions based on NSABP B-39/RTOG 0413 APBI regimen. Although NSABP B-39/RTOG 0413 delivered twice-daily APBI, we chose once-daily APBI because of patient convenience and the limited number of treatment machines. Using the linear quadratic model and assuming an α/β ratio of 4 Gy for breast tumor kill, 10 Gy for acute effects and 3 Gy for late effects, the equivalent dose in 2 Gy fractions (EQD2) doses for this prescription was comparable with other conventionally fractionated and hypofractionated WBI schedules and APBI schedules (Table 2) [5–7]. On the other hand, the present study had slightly higher EQD2 as compared with ultra-hypofractionated WBI regimens represented by the UK FAST-Forward [8, 9].

**Table 2:** Comparing various WBI and APBI fractionation schemes for EQD2(Gy)

	WBI			APBI		
	Conventional fraction	Hypofraction	Ultra-hypofraction			
Study		JCOG0906	FAST Forword	IMPORT LOW	FLORENCE	Present study
Dose(Gy)/fractions	50/25	42.56/16	26/5	40/15	30/5	38.5/10
Tumor control(α/β = 4.0 Gy)	50	47.2	39.9	44.4	50	50
Acute effects(α/β = 10 Gy)	50	44.9	32.9	44.2	40	44.4
Late effects(α/β = 3 Gy)	50	48.2	42.6	45.3	54	52.7

### Sample size

The primary endpoints of this study were the frequency and severity of acute and late radiation toxicities. The incidence of ≥ grade 3 acute and late radiation toxicities when treated with external WBI was considered to be around 3% [10]. Because APBI had the advantage of reduced treatment time and cost compared to WBI, we assumed a threshold incidence of ≥ grade 3 radiation toxicities in APBI of 10% and an expected incidence of 3%. With the type one error rate of 5% and 80% power, 71 patients must be accumulated in this APBI trial. Secondary endpoints were local control, survival, and cosmetic results.

### Follow-up and statistical analysis

Patients were seen first 1 month after the last fraction of APBI, then every 6 months for the first 5 years, and then on an annual basis by the treating radiation oncologist. Annual bilateral mammography or ultrasound was mandatory. Ipsilateral breast tumor recurrence (IBTR) was defined as any histologically confirmed cancer tissue in the treated breast. The follow-up CT scan was not mandatory, so the asymptomatic radiation pneumonitis was detected only by the follow-up chest radiography performed at least annually.

Acute and late radiation toxicities were evaluated and graded according to the Common Terminology Criteria for Adverse Events, version 4 [11]. The values of acute toxicities presented are the patient’s worst toxicity occurring until 3 months after completion of the APBI. Late toxicity was graded by worst toxicity from 4th month after completion of the APBI to the last follow-up visit. The cosmetic outcome was evaluated by a radiation oncologist using the Harvard Scale at 2 years after enrollment because the protocol stipulated a two-year follow-up period [12].

All time intervals were calculated from the starting date of APBI. Time until IBTR was censored on lost follow-up and on death. The cumulative incidence of IBTR, disease free survival (DFS) and overall survival (OS) were calculated using the Kaplan–Meier method. Groups were compared using the log-rank test. For all statistical tests a significance level of 0.05 was used.

## Results

### Patient- and treatment-related characteristics

Between January 2008 and January 2010, 73 patients enrolled in this trial. The patient- and treatment-related characteristics for all eligible patients are shown in Table 3. All patients completed the prescribed course of external beam radiotherapy. Median age was 60 years (range: 32–79 years). In 12 patients (16%), 1–3 lymph nodes metastases were present and all the patients underwent axillary lymph nodes dissection removing at least 8 nodes. Following the ASTRO Consensus Panel (CP) groups, almost half (49%) of patients were classified as ‘suitable’, with ‘cautionary’ and ‘unsuitable’ accounting for 27% and 23% respectively [3, 4]. The median follow-up was 12.6 years (range: 2.7–13.9 years). For 2 patients, late toxicities and cosmetics were not assessed; one patient refused to be followed up by visit and the other patient moved immediately after the APBI, but both of them had been monitored for relapse regularly by telephone.

**Table 3:** Patients and treatment-related characteristics

Characteristics	No
Age (years)	
Median	60
Range	32–79
Tumor laterality	
Right	36 (49%)
Left	37 (51%)
Pathological tumor size (cm)	
Median	1.6
Range	0.2–3.0
Histology	
Invasive ductal	57 (78%)
DCIS	10 (14%)
Mucinous	4 (6%)
Invasive lobular	1 (1%)
Apocrine	1 (1%)
Histologic grade	
1	15 (20%)
2	45 (62%)
3	13 (18%)
Free surgical margin (mm)	
> 5–10	30 (41%)
≥ 10	43 (59%)
Nodal Status	
N0	61 (84%)
pN0 at SLNB	58 (80%)
No nodal assessment because of pure DCIS	3 (4%)
pN1 at ALND	12 (16%)
1 LN metastasis	8 (11%)
2 LN metastases	3 (4%)
3 LN metastases	1 (1%)
Hormone receptor status	
ER +	61 (84%)
PR +	61 (84%)
N/A	1 (1%)
HER2 status	
HER2 +	4 (5%)
Adjuvant treatment	
Hormonal therapy	32 (44%)
Chemotherapy	18 (25%)
Trastuzumab	0 (0%)
ASTRO consensus	
Suitable	36 (49%)
Cautionary	20 (27%)
Unsuitable	17 (23%)
Follow-up(years)	
Median	12.6
Range	2.7–13.9

*DCIS* ductalcarcinoma in situ, *SLNB* sentinel lymph node biopsy, *ALND* axillary lymph node dissection, *LN* lymph node, *ER* estrogen receptor, *PR* progesterone receptor, *N/A* not available, *ASTRO* American society of radiation oncology

### Dosimetric analysis

A total of 73 treatment plans from all the enrolled patients were evaluable for dosimetric analysis. Table 4 lists the results. Due to the smallness of breast volume, V50_ipsi_breast_ (the volume of the ipsilateral breast covered by 50% of the prescribed dose) tended to be much higher than the values of the established constraints.

**Table 4:** Dosimetric analysis

	No. (%)		
No. of beams			
2	25 (34%)		
3	27 (37%)		
4	21 (29%)		
Energy (MV)			
4	40 (55%)		
6	33 (45%)		

	Mean	Median	Minimun-Maximum
**Target volumes**			
PTV_EVAL volume (cm^3^)	124.5	114.0	34–344.8
Ipsilateral total breast volume (cm3)	594.3	562.6	168.3–2055.6
PTV_EVAL/total breast volume (%)	21.6	20.7	8.9–42.1
**PTV parameter**			
D90_PTV_EVAL_ (%)	96.3	96.5	89.5–100
**OAR parameters**			
Ipsilateral breast maximum (%)	107.4	107.0	102.9–115.1
V50_ipsi_breast_ (%)	81.0	85.6	17.5–100
V100_ipsi_breast_ (%)	34.3	32.7	3.4–68.4
Contralateral breast maximum (%)	3.4	1.0	0–54.4
V30_ipsi_lung_ (%)	9.0	7.9	2.8–56.6
V5_contra_lung_ (%)	0.2	0	0–6.4
V5_heart_ (%)			
Right-sided lesion	0.7	0	0–12.8
Left-sided lesion	12.2	8.3	0–63.3

*PTV_EVAL* planning target volume for evaluation, *PTV* planning target volume, *OAR* organ at risk, *ipsi_breast* ipsilateral breast, *ipsi_lung* ipsilateral lung, *contra_lung* contralateral lung, *D90*_*PTV_EVAL*_ the minimun coverage dose of 90% of the PTV_EVAL, *V50, V100*_*ipsi_breast*_ the volume of the ipsilateral breast covered by 50% or 100% of the prescribed dose, *V30*_*ipsi_lung*_ the volume of the ipsilateral lung covered by 30% of the prescribed dose, *V5*_*contra_lung*_ the volume of the contralateral lung covered by 5% of the prescribed dose, *V5*_*heart*_ the volume of the heart covered by 5% of the prescribed dose

### Treatment-related toxicities and cosmetic results

Table 5 lists the acute and late toxicities as well as the cosmetic results. There were no grade 3 or 4 toxicities. A single case of grade 2 radiation pneumonitis was observed 4 months after completion of the APBI, which improved without using steroid. V30ipsi_lung of the case was 14.5% which was below the allowed value of 15%. The most frequent late toxicities observed were skin pigmentation (66.2%) and induration/ fibrosis (64.8%). Grade 2 late toxicity was limited to 1 patient (dry skin). The parameters of the patients were within the set dose-volume constraints.

**Table 5:** Acute and late toxicities and cosmetic results

Toxicity	No. (%)			
	Grade 0	Grade 1	Grade 2	Grade 3–4
**Acute toxicity (n = 73)**				
Fatigue	42 (57.5)	31 (42.5)	0 (0)	0 (0)
Nausea/vomitting	66 (90.4)	7 (9.6)	0 (0)	0 (0)
Anorexia	67 (91.8)	6 (8.2)	0 (0)	0 (0)
Dermatitis	6 (8.2)	67 (91.8)	0 (0)	0 (0)
Dry skin	70 (95.9)	3 (4.1)	0 (0)	0 (0)
Skin pigmentation	55 (75.3)	18 (24.7)	0 (0)	0 (0)
edema	42 (57.5)	31 (42.5)	0 (0)	0 (0)
Breast pain	42 (57.5)	30 (41.1)	1 (1.4)	0 (0)
Subctaneous induration	54 (74)	19 (26)	0 (0)	0 (0)
Pneumonitis	70 (95.9)	2 (2.7)	1 (1.4)	0 (0)
**Late toxicity (n = 71)**				
Dry skin	58 (81.7)	12 (16.9)	1 (1.4)	0 (0)
Skin pigmentation	24 (33.8)	47 (66.2)	0 (0)	0 (0)
Induration/fibrosis	25 (35.2)	46 (64.8)	0 (0)	0 (0)
Telangiectasia	67 (94.4)	4 (5.6)	0 (0)	0 (0)
Breast atrophy	53 (74.6)	18 (25.4)	0 (0)	0 (0)
Breast pain	38 (53.5)	33 (46.5)	0 (0)	0 (0)
**Cosmesis**	**Execellent**	**Good**	**Fair**	**Poor**
At 2 years after enrollment (n = 71)	30 (42.3)	37 (52.1)	4(5.6)	0 (0)
At 10 years after enrollment (n = 28)	9 (32.1)	16 (57.1)	2 (7.1)	1 (3.6)

On the basis of the Harvard Scale for cosmetic outcomes, the majority of patients (94.4%) had excellent/good cosmetic outcomes at 2 years after enrollment (Table 5). A 10-year cosmetic evaluation was performed on 28 patients (38%) of whom 25 (89.3%) had excellent/good cosmetic outcomes and 3 (10.7%) had fair/poor outcomes. All three patients who had fair/poor cosmesis at the 10 year point had fair cosmesis at the 2 year point. Online Resource 3 shows the transition of cosmetic outcomes, which were compared between 2 and 10 years after enrollment.

### Treatment efficacy

Twelve-year outcomes are presented in Table 6. Four patients (5.5%) developed IBTR 2.9, 7.9, 11.4 and 13 years after APBI. The first two IBTR occurred as isolated cutaneous relapse in the irradiated field and were found on inspection. One patient underwent mastectomy of ipsilateral breast as a salvage treatment. The other patient has received chemotherapy as a salvage treatment because the cutaneous erythema had invaded from ipsilateral breast to the medial part of contralateral breast, and the histological subtype of this new cancer was ER-, PR- and HER2 + , while the first cancer was ER + , PR + and HER2-. The latter two IBTR occurred in different quadrants of the breast from the first cancer and both of them underwent mastectomy. The 10- and 12-year cumulative incidence of IBTR was 2.8% and 4.4%.

**Table 6:** Twelve-year treatment outcomes by age and nodal status

	At 12 y No. (cumulative incidence)						
	All patients	age ≥ 50	age < 50	*p*	Node-negative	Node-positive	*p*
	(n = 73)	(n = 56)	(n = 17)		(n = 61)	(n = 12)	
IBTR	3 (4.4%)	2 (3.6%)	1 (7.7%)	0.81	1 (1.9%)	2 (16.7%)	0.055
Regional nodal failure	1 (1.4%)	1 (1.8%)	0	0.58	1 (1.6%)	0	0.66
Distant failure	3 (4.3%)	3 (5.5%)	0	0.35	2 (3.3%)	1(9.1%)	0.45
Disease free survival	91.5%	91.0%	92.3%	0.63	93.1%	83.3%	0.36
Overall Survival	94.3%	92.7%	100.0%	0.69	93.2%	100.0%	0.82

*IBTR* ipsilateral breast tumor recurrence

There were 1 regional nodal failure at 1 year after APBI (12-year cumulative incidence: 1.4%) and 3 distant failure at 4.1, 4.8 and 10.8 years after APBI (12-year cumulative incidence: 4.3%). In 4 patients, contralateral breast cancer occurred (12-year cumulative incidence: 5.6%). During the follow-up, 3 died from recurrence of the breast cancer and 4 died of causes other than the breast cancer: 1 of the four died from contralateral breast cancer. Of the 4 patients with IBTR, only one with distant metastasis died. The 10-year point estimate for DFS and OS was 93.1% and 97.2%, and the 12 year point estimate for DFS and OS was 91.5% and 94.3%, respectively.

Two of the four IBTR cases were detected among the ASTRO CP ‘unsuitable’ group and the decisive factors on ‘unsuitable’ group in the 2 IBTR cases were not age but positive axillary nodes. The difference in the rates of IBTR between node-negative and node-positive patients was marginal (12 year cumulative incidence of IBTR: 1.9% vs. 16.7%, p = 0.055 log-rank, p = 0.016 Gehan-Breslow-Wilcoxon) (Table 6). No significant difference was found in the 12 year point estimate for DFS and OS between age ≥ 50 and age < 50 or between node-negative and node-positive patients. Online Resource 2 lists the 12 year treatment outcomes for each ASTRO CP group.

## Discussion

To date, four groups published their 10 year results of APBI with EBRT (Table 7) [7, 13–18]. Although many long-term results of APBI with brachytherapy have been reported, there are few reports of APBI with EBRT exceeding 10 years of follow-up. This prospective study targeting to Japanese breast cancer patients showed comparable results to other APBI series with regard to long-term local tumor control, toxicity, and cosmetic results.

**Table 7:** Comparison of published series of prospective APBI studies using EBRT techniques (with a minimun follow-up of 10 years)

Study/Institute	Year	n	Age restriction	Node Positive	technique	Fractionation sheme	Mean PTV volume (cm^3^)	Mean ipsilateral breast volume (cm3)	Mean PTV_EVAL/breast volume (%)	Median FUP (years)	IBTR (%)	≥ G3 toxicity (%)	Excellent/good cosmesis (%)
University of Florence [7]	2020	260	≥ 40	Yes	IMRT	6 Gy × 5	123*	NR	21*	10.5	3.7 (10-year)	0	100
NSABP B-39/RTOG 0413 [14]	2019	2107	≥ 18	Yes	3D-CRT or brachytherapy)	3.85 Gy × 10	NR	NR	NR	10.2	4.6 (10-year)	10	NR
Universidad Autónoma de Barcelona [13]	2021	51	≥ 60	No	3D-CRT	3.75 Gy × 10	255.8**	1046**	24**	10.3	2	0	84.1
Rutgers Cancer Institute of New Jersey [15]	2024	45	≥ 45	No	3D-CRT	3.33 Gy × 15	301.2***	1707.1***	17.6***	12	5.8	2.9	91
Present study	2024	73	≥ 20	Yes	3D-CRT	3.85 Gy × 10	124.5	594.3	21.6	12.6	2.8 (10-year)	0	94.4 (at 2-year) 89.3 (at 10-year)

*EBRT* external beam radiation therapy, *APBI* accelerated partial breast irradiation, *PTV* planning target volume, *FUP* folow-up period, *IBTR* ipsilateral breast tumor recurrence, *3D-CRT* three-dimensional conformal radiation therapy, *IMRT* intensity modulated radiation therapy, *NR* not reported

*Data reported by Livi et al.[16]

**Data reported by Rodriguez et al.[17]

***Data reported by Goyal et al.[18]

The eligibility criteria of our study included patients of young age and patients with positive axillary nodes because the criteria were based on the NSABP B-39/RTOG 0413. They were unsuitable characteristics according to present inclusion criteria for APBI: the ASTRO CP guidelines on the off-protocol use of APBI listed < 50 year-old patients in the ‘cautionary’ or ‘unsuitable’ and node-positive patients in the ‘unsuitable’ category [3, 4]. This study began registration with approval from the ethics committee in October 2007, before the 2009 ASTRO guidelines were published. Therefore, during the planning of the study, the safety of the eligibility criteria was not assessed in consideration of the ASTRO suitability group. Like the NSABP B-39/RTOG 0413 trial, this study aimed to assess treatment efficacy for the population including subgroups known to have both worse and better IBTR incidence that is broadly representative of all breast cancer patients undergoing radiotherapy after lumpectomy.

In our trial, 17 patients (23.3%) were below the age of 50, but no significant difference was detected in the 12 year point estimate of IBTR, regional nodal failure, distant failure, DFS and OS between ≥ 50 and < 50 year-old patients. On the other hand, the rate of IBTR were marginally higher in the patients with positive lymph nodes than those with negative lymph nodes. As with ASTRO CP, the guidelines developed by other groups also do not recommend young or node-positive patients to be treated using APBI, except in a clinical trial [19, 20]. In NSABP B-39/RTOG 0413, 38% were under the age of 50, and pre- and post-menopausal subgroups did not identify significant differences between the IBTR of APBI and WBI. Up to three axillary lymph nodes could be positive in the study, with DCIS or pN0 accounting for 90% in both groups. Subgroup analyses of IBTR for DCIS, N0, and N1 showed no significant differences in IBTR between APBI and WBI [14]. However, limited research has been completed to determine the ideal age criteria for patients undergoing APBI, and only limited data remains available on patients with node-positive disease treated with APBI despite node-positive patients being included in some trials [14, 21, 22]. Our findings might support references that suggest APBI is a feasible treatment option for young breast cancer patients, and off-protocol patients should be node-negative.

Although NSABP B-39/RTOG 0413 delivered twice-daily APBI, we chose once-daily APBI for patient convenience and the limited number of treatment machines. Hoopes et al. reported that 70% of women prefer once-daily radiation over 10 days vs. twice-daily radiation over 5 days [23]. Because NSABP B-39/RTOG 0413 included all APBI modalities (twice-daily EBRT or brachytherapy) as one group, it was unclear whether acute and late toxicities differed between patients receiving different APBI methods [14]. The RAPID trial, which used the same twice-daily APBI regimen (38.5 Gy in 10 fractions over 5 treatment days), showed that acute toxicity was less with twice-daily APBI, while grade ≥ 2 late toxicity was increased with twice-daily APBI (32% vs. 13%) [22]. The increased 7 year fair/poor cosmesis (36% vs. 19%) with APBI was likely caused by the twice-daily fractionation. Following the RAPID results, the recently published ASTRO guidelines do not recommend twice-daily APBI with EBRT with respect to toxicity and cosmesis [24]. Consistent with the RAPID study, the IRMA trial, which randomly assigned over 3300 patients to receive twice-daily APBI of 38.5 Gy in 10 fractions or WBI, showed that twice-daily APBI had higher rates of late soft tissue toxicity (2.8% vs. 1%), bone toxicity (1.1% vs. 0%), and adverse cosmesis [25]. The randomized trial by Boutrus et al. [26] compared once-daily APBI vs. twice-daily APBI schemes with EBRT (38.5 Gy in 10 fractions) on 113 patients. The once-daily APBI arm had similar local control and acute toxicities, and there was a statistically significant decrease in late skin toxicity (3.8% vs. 11.7%). The 2 year fair/poor cosmesis was lower for once-daily APBI fractionation than for twice-daily APBI fractionation (7.5% vs. 26.7%). The long-term results of our study support the favorable results of once-daily APBI with EBRT.

Ipsilateral breast volume of this study was obviously smaller compared to those of other APBI trials (Table 7). Generally, small breasts are considered unsuitable for APBI because the PTV tend to be close to the risk organ including skin, chest wall and lung, resulting excessive exposure of normal tissue. In the cases of APBI with small breasts, the ratio of the PTV to the ipsilateral breast volume would be inescapably larger than the cases of large breasts. Hepel et al. [27] reported that PTV_EVAL/total breast volume was found to be correlated with both grade 2–4 fibrosis and fair/poor cosmesis; the mean PTV_EVAL/total breast volume was 19% for all patients, 18% for patients with excellent/good cosmesis, and 24% for patients with fair/poor cosmesis. Shaitelman et al. [28] found a small cavity-to-skin distance was associated with an increased risk of grade ≥ 2 induration, a marginally increased risk of grade ≥ 2 volume reduction, and poor or fair cosmesis.

APBI delivered with multicatheter brachytherapy permit better dose distribution with favorable dose concentration and without excessive exposure of normal tissue even in the small breasts. The 5 year results of a multi-institutional prospective study of APBI with multicatheter brachytherapy for Japanese patients were published [29]. There were no reports of Grade 4 late toxicity, local recurrence, or breast cancer-related deaths. The overall score for cosmetic outcome was 74% Excellent/Good. Although the optimal method of APBI for small breasts is still unclear, our study demonstrated that APBI with 3DCRT is a feasible option for Asian females with small breasts in terms of toxicity and cosmetics.

One benefit of APBI over WBI is the possibility of a shorter irradiation period. In this study, there were many cases where the majority of the breast was irradiated due to the small breast volume, and as a result, they were similar to cases of hypofractionated WBI with slightly lower doses. In recent years, there has been a lot of research into reducing the period of WBI. The UK FAST-Forward trial proved the non-inferiority of ultra-hypofractionated-WBI (26 Gy in five fractions over one week) to the standard 40 Gy in 15 fractions over three weeks, in terms of local tumor control and clinician-assessed effects on normal tissues [8, 9]. A multi-institutional study of ultra-hypofractionated WBI is currently ongoing also in Japan [30]. In terms of the treatment period, in 2007 when this study was planned, there was a significant advantage to APBI which could be treated in 10 fractions, greatly shortening the 25–30 fractions of conventional WBI. But in recent years, there has been almost no difference in the treatment period between WBI and APBI. However, further treatment period reductions have recently been considered. There are limits to the adaptation of hypofractionation when the irradiation volume is large as in the case of WBI. The ultra-APBI regimen, which reduces irradiation days to three or less, has been proposed, and it has demonstrated positive outcomes on adverse events and local control in low-risk early-stage breast cancer [31, 32].

The major limitation of this study is that it was not possible to rule out the impact of surgery on the cosmetic outcome or assess the changes caused by APBI alone because the cosmetic evaluation had not been carried out before irradiation. As another limitation, we could not assess the cosmesis of all patients over a 10-year period. However, although the number of cases is limited, the 10-year cosmesis was good, with 89.3% of patients obtaining an excellent/good evaluation. Additionally, no cases of significant cosmetic deterioration were found when comparing the appearance at 2 and 10 years. Notably, the apparent breast asymmetry of patients with fair/poor cosmesis was considered Grade 1 breast atrophy since it was a postoperative change rather than an atrophy brought on by irradiation. Another limitation is that the Harvard Scale was scored only by physicians, and cosmesis was not assessed by patients themselves. If there might be discrepancies in the cosmetic assessments between the physicians and patients, then the cosmetic outcomes of this study should be interpreted with caution. However, the strengths of our study include that the median follow-up length of 12 years was sufficiently long to take into account the latency of radiation toxicities, and this is the first report of a long follow-up period of ≥ 10 years of once-daily APBI with EBRT from Asian countries.

## Conclusions

The local tumor control, toxicity, and cosmesis of once-daily APBI delivered with 3D-CRT for Japanese females were satisfactory and compatible with the long-term outcomes of other ABPI trials. This prospective study demonstrated that once-daily APBI delivered with 3D-CRT is a feasible treatment option alternative to WBI for early-stage breast cancer patients, even in Asian countries, but also indicated that node-positive status might increase IBTR risk.

## Supplementary Information

Below is the link to the electronic supplementary material.

Supplementary file1 (DOCX 37 KB)

Supplementary file2 (DOCX 37 KB)

Supplementary file3 (DOCX 425 KB)

## Data Availability

The dataset generated and analyzed during the current study are available from the corresponding author on reasonable request.
