# Supplementary material for: Prospective study of once-daily accelerated partial breast irradiation using 3-dimensional conformal external beam radiotherapy for Japanese women: 12-year outcomes, toxicity, and cosmesis
Source: Breast Cancer. 2024 Dec 4;32(1):197–207. doi: 10.1007/s12282-024-01650-x (PMC11717833; doi:10.1007/s12282-024-01650-x)
Supplement: Supplementary file 1 — Supplementary file1 (DOCX 37 KB) [file 12282_2024_1650_MOESM1_ESM.docx]

**Prospective study of once-daily accelerated partial breast irradiation using 3-dimensional conformal external beam radiotherapy for Japanese women: 12-year outcomes, toxicity, and cosmesis**

**Journal: *Breast Cancer***

Kana Takahashi, Yoshikazu Kagami, Ryoichi Yoshimura, Madoka Morota, Naoya Murakami, Satoshi Nakamura, Hiroyuki Okamoto, Ayaka Nagao, Madoka Sakuramachi, Tairo Kashihara, Tomoya Kaneda, Koji Inaba, Kae Okuma, Yuko Nakayama, Jun Itami, Hiroshi Igaki

Corresponding author email: kantakah@ncc.go.jp
